# Mechanical loading of bioengineered skeletal muscle in vitro recapitulates gene expression signatures of resistance exercise in vivo

**DOI:** 10.1002/jcp.30328

**Published:** 2021-02-15

**Authors:** Daniel C. Turner, Piotr P. Gorski, Robert A. Seaborne, Mark Viggars, Mark Murphy, Jonathan C. Jarvis, Neil R.W. Martin, Claire E. Stewart, Adam P. Sharples

**Affiliations:** ^1^ Institute for Science and Technology in Medicine (ISTM), School of Pharmacy and Bioengineering Keele University Staffordshire UK; ^2^ Stem Cells, Ageing and Molecular Physiology Unit (SCAMP), Exercise Metabolism and Adaptation Research Group (EMARG), Research Institute for Sport and Exercise Sciences (RISES) Liverpool John Moores University Liverpool UK; ^3^ Randall Centre for Cell and Molecular Biophysics, School of Basic and Medical Biosciences King's College London London UK; ^4^ Institute for Physical Performance Norwegian School of Sport Sciences (NiH) Oslo Norway; ^5^ Center for Genomics and Child Health, Blizard Institute, Barts and the London School of Medicine and Dentistry Queen Mary University of London London UK; ^6^ School of Pharmacy and Biomolecular Sciences Liverpool John Moores University Liverpool UK; ^7^ School of Sport, Exercise and Health Sciences Loughborough University Loughborough UK

**Keywords:** bioengineering, DNA methylation, fibrin, gene expression, mechanical loading, skeletal muscle

## Abstract

Understanding the role of mechanical loading and exercise in skeletal muscle (SkM) is paramount for delineating the molecular mechanisms that govern changes in muscle mass. However, it is unknown whether loading of bioengineered SkM in vitro adequately recapitulates the molecular responses observed after resistance exercise (RE) in vivo. To address this, the transcriptional and epigenetic (DNA methylation) responses were compared after mechanical loading in bioengineered SkM in vitro and after RE in vivo. Specifically, genes known to be upregulated/hypomethylated after RE in humans were analyzed. Ninety‐three percent of these genes demonstrated similar changes in gene expression post‐loading in the bioengineered muscle when compared to acute RE in humans. Furthermore, similar differences in gene expression were observed between loaded bioengineered SkM and after programmed RT in rat SkM tissue. Hypomethylation occurred for only one of the genes analysed (GRIK2) post‐loading in bioengineered SkM. To further validate these findings, DNA methylation and mRNA expression of known hypomethylated and upregulated genes post‐acute RE in humans were also analyzed at 0.5, 3, and 24 h post‐loading in bioengineered muscle. The largest changes in gene expression occurred at 3 h, whereby 82% and 91% of genes responded similarly when compared to human and rodent SkM respectively. DNA methylation of only a small proportion of genes analyzed (TRAF1, MSN, and CTTN) significantly increased post‐loading in bioengineered SkM alone. Overall, mechanical loading of bioengineered SkM in vitro recapitulates the gene expression profile of human and rodent SkM after RE in vivo. Although some genes demonstrated differential DNA methylation post‐loading in bioengineered SkM, such changes across the majority of genes analyzed did not closely mimic the epigenetic response to acute‐RE in humans.

## Graphical abstract

Mechanical loading of mouse bioengineered skeletal muscle in vitro recapitulates the gene expression profile of skeletal muscle after resistance exercise in vivo. However, while some DNA methylation changes were detected following mechanical loading in bioengineered muscle, this did not as closely mimic the DNA methylation response to acute resistance exercise in vivo.

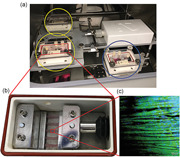

## 1 INTRODUCTION

Skeletal muscle (SkM) is a highly abundant and mechano‐sensitive tissue, displaying functional and morphological changes in the presence or absence of loading. Indeed, muscle size and strength increase in response to resistance exercise (RE) or mechanical loading (Baar & Esser, 1999; Baehr et al., 2014; Bodine et al., 2001; Goldberg & Goodman, 1969; Goldberg, 1967, 1968; Goodman et al., 2011; Schiaffino et al., 1972, 1976; Seaborne et al., 2018b). In contrast, SkM mass declines and function is compromised during periods of unloading, such as during disuse (Baehr et al., 2017; Seaborne et al., 2018a, 2018b, 2019; Wall et al., 2014), spaceflight (Edgerton et al., 1995), and bed rest (Dirks et al., 2016). Relative inactivity is also an important contributor to age‐related muscle loss (Hughes et al., 2001; Morse et al., 2005).

Despite the well‐established role of transcription in regulating gene expression during load‐induced SkM hypertrophy (Goldberg & Goodman, 1969), the importance of epigenetic changes, specifically DNA methylation, has more recently emerged (Seaborne et al., 2018b, 2018a; Turner et al., 2019b). DNA methylation is a common epigenetic modification and is characterized by the addition (hypermethylation) or removal (hypomethylation) of covalent methyl groups on cytosine nucleotides within cytosine‐guanidine base pairings (CpG sites) (Bird, 1986). Such modifications within the promoter or enhancer regions of coding genes generally tend to permit (via hypomethylation) or prevent (via hypermethylation) gene transcription, and thereby closely regulate gene expression (Bogdanović & Veenstra, 2009). However, gene expression is not exclusively regulated by DNA methylation within promoter or enhancer regions as there is also a correlation between gene body methylation and gene transcription (Anastasiadi et al., 2018; Ball et al., 2009; Brenet et al., 2011). Recent work demonstrated differential genome‐wide (850K CpG sites) DNA methylation changes after acute and chronic RE in humans, and following periods of detraining and retraining (Seaborne et al., 2018b, 2018a). Interestingly, specific gene clusters were epigenetically altered after a single bout of RE, and displayed significant hypomethylated signatures that were retained even throughout detraining with increased hypomethylation and enhanced gene expression after retraining (Seaborne et al., 2018b, 2018a). Suggestive that the epigenetic modification of these genes represented an epigenetic memory of earlier training. More recent work mapped the methylome data derived from Seaborne et al., (2018b, 2018a) against publicly available transcriptomic data sets obtained from 110 acute and 181 chronic RE studies in humans to determine whether epigenetically regulated genes across the methylome were also differentially regulated across the human transcriptome after RE (Turner et al., 2019b). Interestingly, several genes enriched in growth‐related pathways, including focal adhesion, mitogen‐activated protein kinase signaling, PI3K‐Akt‐mTOR signaling, p53 signaling, Jak‐STAT signaling, tumor growth factor β (TGF‐β), and notch signaling, demonstrated a distinct inverse relationship between DNA methylation and gene expression, supporting the notion that a single bout of exercise is able to evoke significant epigenetic modifications that correspond with significant changes in expression of the same genes.

The molecular responses following mechanical loading in engineered tissues are typically assessed at the messenger RNA (mRNA) expression (Aguilar‐Agon et al., 2020; Cheema et al., 2005; Heher et al., 2015; Mudera et al., 2000; Player et al., 2014; Sawadkar et al., 2019; Verhoekx et al., 2013) and more recently, protein phosphorylation levels (Aguilar‐Agon et al., 2019). Such investigations have been restricted to studying well‐characterized mechano‐sensitive genes and proteins. Indeed, there is a paucity of data using similar in vitro models that may assist in characterizing the epigenetic and transcriptional changes observed across the methylome and transcriptome following exercise in human SkM. At the candidate gene level, work by our group mechanically loaded C_2_C_12_ fibrin bioengineered SkM to determine the response of the recently characterized E3 ubiquitin ligase, UBR5 (Seaborne et al., 2019), that was first identified in SkM after exercise in humans (Seaborne et al., 2018a, 2018b). Interestingly, UBR5 mRNA expression increased to a similar extent following loading in mouse bioengineered SkM (~1.6‐fold) and after an acute bout of RE in humans (~1.7‐fold) (Seaborne et al., 2018b, 2018a, 2019). Despite such promising findings, there has been no previous attempt to study the load‐induced epigenetic and associated transcriptional responses in bioengineered SkM and whether changes resemble comparable epigenetic and transcriptional profiles observed after RE in humans. Such experiments are essential to determine whether mechanical loading of bioengineered SkM provides a relevant in vitro model for studying the mechanisms underpinning load‐induced SkM anabolism and hypertrophy that occur in vivo.

The objectives of the present study were, therefore, to assess whether mechanical loading of bioengineered SkM using commercially available mouse C_2_C_12_ cells mimics the DNA methylation and gene expression signatures that we have previously identified across the methylome (Seaborne et al., 2018b, 2018a) and across both the methylome and transcriptome (Turner et al., 2019b) after RE in humans. Such experiments would validate (or invalidate) mechanical loading of C_2_C_12_ bioengineered muscle as a representative in vitro model of RE in vivo, at least at the gene transcription and DNA methylation level. Given that DNA methylation and gene expression are altered after a single bout of resistance (Seaborne et al., 2018b, 2018a) or endurance (Barrès et al., 2012) exercise in vivo, it was hypothesized that acute mechanical loading of fibrin‐bioengineered SkM would evoke methylation and transcriptional changes that would mimic the responses identified after RE in vivo.

## 2 METHODS

### 2.1 Monolayer cell culture

Murine C_2_C_12_ SkM myoblasts (Blau et al., 1985; Yaffe & Saxel, 1977) were first seeded (1 × 10^6^ cells) onto pre‐gelatinized (0.2% in dH_2_O; Type A; Sigma‐Aldrich) T75 flasks (Nunc™; Thermo Fisher Scientific) within a Class II biological safety cabinet (BSC; Kojair) and expanded in growth media composed of high glucose (4.5 g/L) Dulbecco's modified Eagle's medium, including 4 mM *l* ‐Glutamine (LG; Sigma‐Aldrich), 10% heat‐inactivated fetal bovine serum (hiFBS; SLS), 10% heat‐inactivated newborn calf serum (hiNBCS; Fisher Scientific, Denmark), supplemented with an additional 2 mM LG (Lonza), 100 U/ml penicillin‐100 μg/ml streptomycin (PS; Lonza) in a humidified incubator (HERAcell 150i; Thermo Scientific) at 37°C, 5% CO_2_ until 80% confluency was attained. Once confluent, cells were washed twice with sterile phosphate‐buffered saline (1× PBS; Sigma‐Aldrich), trypsinized (0.05% Trypsin/0.02% EDT; Sigma‐Aldrich), and counted using the trypan blue exclusion method (0.4% trypan blue; Sigma‐Aldrich).

### 2.2 Bioengineering of murine fibrin skeletal muscle

**Figure 1:**
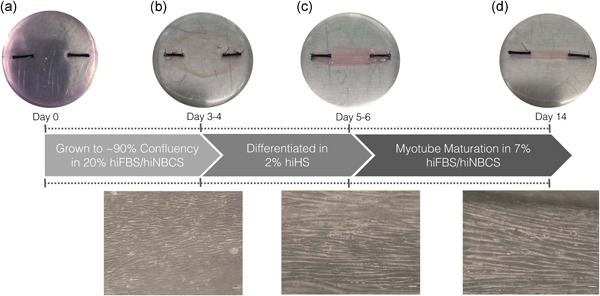
Schematic representation of procedures for bioengineering fibrin mouse skeletal muscle (SkM). Macro‐ and microscopic images of C_2_C_12_ fibrin bioengineered muscle at (a) 0 days, (b) 3–4 days grown to confluency in 20% heat‐inactivated fetal bovine serum (hiFBS)/heat‐inactivated newborn calf serum (hiNBCS), (c) 5–6 days differentiated in heat‐inactivated horse serum (hiHS) for 48 h and (d) myotubes matured up to 14 days in 7% hiFBS/hiNBCS (10× magnification, scale bar = 50 μm, Olympus, CKX31)

Murine C_2_C_12_ bioengineered SkM was prepared as previously described in detail elsewhere (Khodabukus & Baar, 2009; Martin et al., 2013; Seaborne et al., 2019; Turner et al., 2019a). Briefly, 2 × 6 mm silk suture threads (Ethicon Mersilk, 2.0) were pinned 12 mm apart using 0.15 mm Minutien pins (Entomoravia) within sylgard‐coated (Sylgard™ 184 Elastomer Kit; Dow Corning) 35 mm culture dishes (Easy‐Grip, BD Falcon®; VWR). Culture dishes were filled with 70% ethanol and left to air dry under UV (programmed to 1 h) overnight in a Class II BSC. Once sterilized, 500 μl GM containing 10 U/ml thrombin (T4648; Sigma‐Aldrich), 8 μl/ml aprotinin (10 mg/ml; A3428; Sigma‐Aldrich), and 0.5 mg/ml 6‐aminocaproic acid (6AA) were added to each culture dish and agitated to ensure the entire surface was covered. Two‐hundred microliters of fibrinogen (20 mg/ml; F8630; Sigma‐Aldrich) were added dropwise and left to incubate at room temperature (RT) for 10 min before transferring to an incubator (37°C, 5% CO_2_) for 1 h to polymerize. Following polymerization, C_2_C_12_ cells were seeded onto the fibrin gel at a concentration of 9 × 10^4^ cells/ml in 2 ml GM containing 0.5 mg/ml 6AA and 50 μM of both *l* ‐ascorbic acid (A4403; Sigma‐Aldrich) and *l* ‐proline (LP; P8865; Sigma‐Aldrich). GM was changed every 48 h until cells were approximately 90% confluent, at which point the media was switched to differentiation media (DM; same composition as GM with the exception of increased 6AA to 1 mg/ml and reduced serum to 2% using heat‐inactivated horse serum, hiHS; Figure 1). Following 48 h in DM, fibrin gels were washed 2 × PBS and media was changed to maintenance medium (MM; same components as GM with reduced 3.5% hiFBS and 3.5% hiNBCS serum and increased 1 mg/ml 6AA). MM was changed every 48 h and a 0.5 ml top‐up of MM was provided on days where media was not changed until constructs had matured into cylindrical‐like muscles with uniaxial myotubes by day 14 (Figure 1d). At day 14, construct width was assessed using digital Vernier calipers (all displaying <4 mm at the narrowest point of the construct) for all constructs used for loading experiments (Khodabukus & Baar, 2009, 2012, 2015a, 2015b; Khodabukus et al., 2015).

### 2.3 Mechanical loading of bioengineered SKM

**Figure 2:**
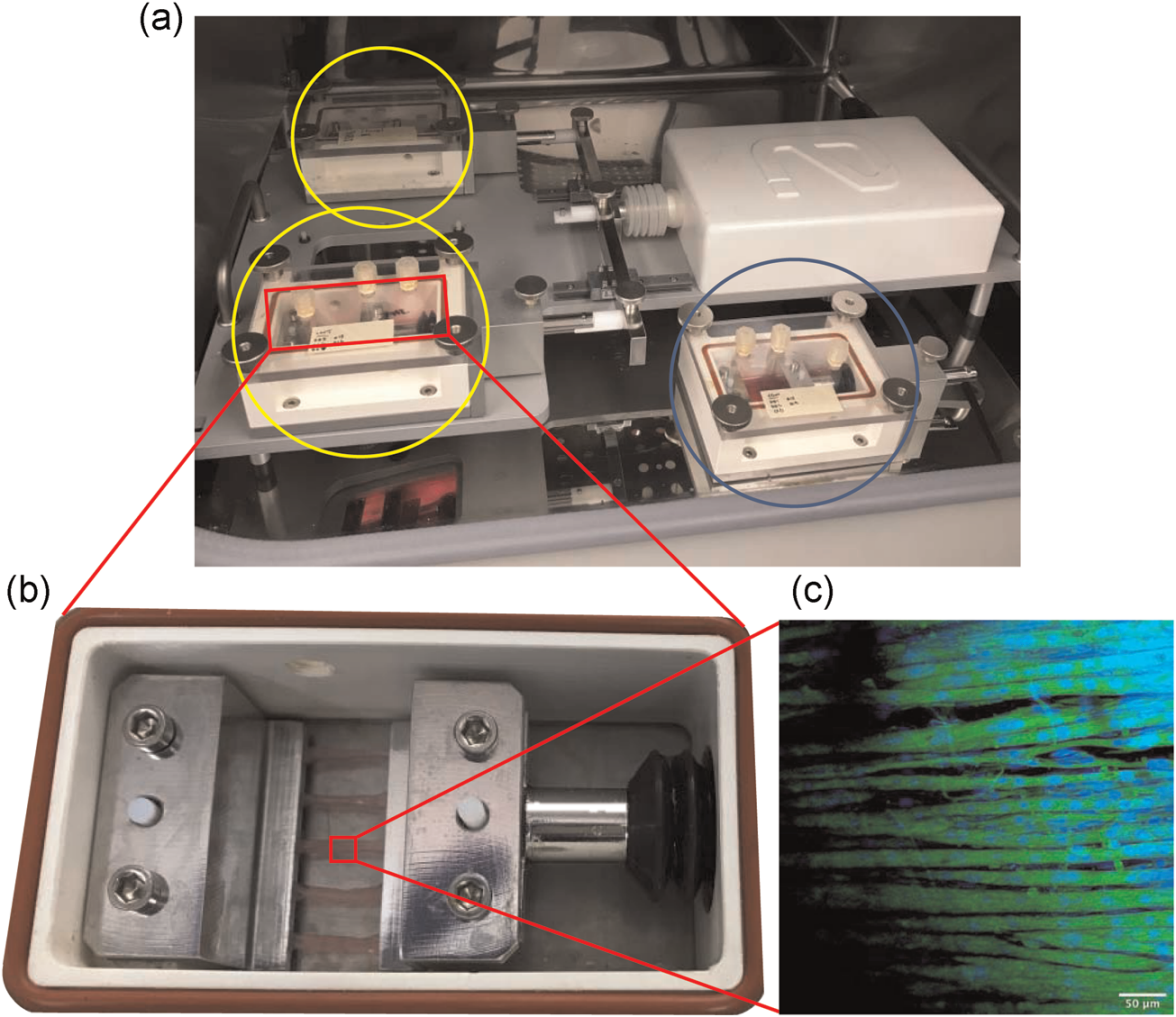
Diagrammatic representation of mechanical loading of mouse bioengineered SkM. (a) The TC‐3 bioreactor system is used to mechanically load bioengineered SkM. Bioreactor chambers were either assembled to the mechanical loading unit (loaded, YELLOW circles) or placed next to the bioreactor (non‐loaded, BLUE circle) in a humidified incubator at 37°C/5% CO_2_. (b) 5 × C_2_C_12_ fibrin‐bioengineered SkM constructs clamped within a single bioreactor chamber. (c) Microscopic image was taken of a muscle construct immunostained for f‐actin (phalloidin‐FITC, green) and myonuclei (DAPI, blue) and imaged using confocal microscopy (Olympus IX83, Japan; 20×, scale bar = 50 μm). The same loaded bioengineered muscle samples were utilized to assess UBR5 gene expression in Seaborne et al., (2019). Therefore, the immuno‐image is taken from Seaborne et al., (2019). *Journal of Physiology* (Wiley), 597.14 (2019) pp 3727–3749, with permission (Copyright‐2019) from the authors. *The Journal of Physiology* (Copyright‐2019 The Physiological Society). DAPI, 4′,6‐diamidino‐2‐phenylindole; FITC, fluorescein isothiocyanate; SkM, skeletal muscle

The TC‐3 tension bioreactor system (EBERS Medical Technology) was used to mechanically load bioengineered SkM constructs (Figure 2a). After 14 days in culture, bioengineered muscle constructs displaying healthy aligned myotubes (Figure 1d) that also spontaneously twitch in situ (Video ‐ Supplementary File 1) were randomly selected for both loaded (*n* = 4–5 replicate cultures) and non‐loaded (*n* = 4–5 replicate cultures) conditions. Constructs were transferred to the bioreactor chambers and submerged in 20 ml MM (Figure 2b). Chambers were attached to the bioreactor system, housed in a humidified incubator (37°C, 5% CO_2_) ready to undergo mechanical loading. Non‐loaded controls were kept at resting length (12 mm) for 1 h. Loaded constructs were subject to 10% (1.2 mm) stretch (which increases myotube hypertrophy, myoblast differentiation, and candidate anabolic gene expression in bioengineered SkM; Heher et al., 2015; Player et al., 2014; Powell et al., 2002) for 4 sets × 10 repetitions (frequency of 0.3 Hz, 0.4 mm/s). Each set was interspersed with 90 s rest and repeated five times. Every 4 sets of 10 repetitions were separated by 3.5 min rest, totaling an intermittent regime over 1 h, matching the non‐loaded control duration. Following the cessation of mechanical loading, constructs were kept at resting length (12 mm) and sampled at 0.5, 3, and 24 h after loading.

### 2.4 Immunohistochemistry and microscopy

Bioengineered SkM muscle constructs were stained and imaged as previously described (Seaborne et al., 2019). Briefly, on day 14, muscle constructs were washed 3 × in Tris‐buffered saline (TBS; 1×; Sigma‐Aldrich) and fixed using ice‐cold methanol:acetone:TBS (25:25:50) for 15 min, and then a further 15 min in methanol:acetone (50:50) only. After a further 3 × washes, culture dishes were wrapped in parafilm and stored at 4°C until required for immunostaining. Following fixation, pins were removed, and constructs were transferred to 2 ml Eppendorf tubes using angled forceps. Bioengineered muscle samples were then permeabilized (0.2% Triton X‐100) and blocked (5% goat serum) in TBS (1×) for 1.5 h and incubated overnight (4°C) in 250 μl of phalloidin‐fluorescein isothiocyanate (FITC) antibody (P5282; Sigma‐Aldrich) at a concentration of 50 μg/ml. After overnight incubation, the secondary antibody was aspirated, and gels were washed 3 × in TBS Before adding 250 μl of 4′,6‐diamidino‐2‐phenylindole (DAPI; 300 nM) for 90 min to counterstain myonuclei (Figure 2c). Once stained, muscle constructs were transferred to nonsylgard‐coated culture dishes containing 2 ml of TBS and were wrapped in parafilm and foil, then stored at 4°C until required for fluorescence imaging. Immunostained constructs were visualized using a confocal fluorescence microscope (Olympus IX83) and imaged using supporting software (FV10‐ASW 4.2; Olympus) to illustrate the alignment of mature myotubes via detection of f‐actin (phalloidin‐FITC, green) and myonuclei (DAPI, blue; Figure 2c).

### 2.5 RNA extraction, primer design, and polymerase chain reaction

Following the cessation of mechanical loading, bioengineered muscle constructs were removed from the bioreactor chambers at 0.5, 3, and 24 h post‐loading. Both loaded and non‐loaded muscle constructs were transferred to MagNA Lyser Green Bead tubes (Roche) containing either 1 ml TRIzol (Invitrogen™; Thermo Fisher Scientific) or 600 μl of Buffer RLT (AllPrep RNA/DNA Mini Kit; Qiagen) containing 6 μl β‐Mercaptoethanol (Sigma‐Aldrich) when isolating RNA using the TRIzol (for analyzing genes identified in Seaborne et al., 2018b, 2018a) or AllPrep RNA/DNA Mini Kit (for analyzing genes identified in Turner et al., 2019b) methods, respectively. Samples were transferred to a MagNA Lyser instrument (Roche, MagNA lyser) and were homogenized (45 s at 6000 rpm, repeated 3× with samples placed on ice for 5 min after each disruption). Concentrations were quantified using spectrophotometry (NanoDrop™ 2000; Thermo Fisher Scientific). A one‐step PCR kit (QuantiFast™ SYBR® Green; Qiagen) was used to assess gene expression. Samples were first diluted in nuclease‐free H_2_O to ensure a concentration of 35 ng RNA in 10 μl volume, made up of 4.75 μl (7.37 ng/μl) RNA sample and 5.25 μl of master mix (MM) composed of 5 μl SYBR green, 0.1 μl of reverse transcriptase (RT) and 0.075 μl of both forward and reverse primers (both 100 µM stock concentration) in polymerase chain reaction (PCR) reaction tubes (0.1 ml strips and caps; Qiagen). Primers were designed using Basic Local Alignment Search Tool (BLAST; http://blast.ncbi.nlm.nih.gov/) and Clustal Omega (https://www.ebi.ac.uk/Tools/msa/clustalo/) to identify gene regions, which shared the same sequence across all transcript variants. Specificity was confirmed via BLAST and melt curve analysis. Primers were purchased from Sigma‐Aldrich. All primer sequence and location information are detailed in Supplementary File 2. PCR amplification was performed using a quantitative reverse transcription‐PCR thermal cycler (Rotorgene 3000Q; Qiagen) and the following protocol: 10 min hold at 50°C (reverse transcription/cDNA synthesis), 95°C for 5 min (transcriptase inactivation and initial denaturation step) and PCR Steps of 40 cycles; 95°C for 10 s (denaturation), 60°C for 30 s (annealing and extension). Upon completion, melt curve analyses confirmed that only one gene product (i.e., the gene of interest) was amplified. Gene expression was determined using the ^ΔΔ^ *C*_t_ equation (Schmittgen & Livak, 2008). The pooled C_T_ values from the non‐loaded controls at each individual time point (0.5, 3, and 24 h) were used as the calibrator condition and were relativized to the mean *C*_t_ value of the reference gene, RP‐IIβ (18.64 ± 0.74, with a low 3.97% variation). PCR efficiencies were similar for the reference gene (RP‐IIβ, 92.24 ± 5.43%, variation 5.88%) and genes of interest (93.64 ± 5.91%, variation 6.31%).

### 2.6 DNA isolation, bisulfite conversion, and targeted DNA methylation

The DNeasy Blood and Tissue Kit (Qiagen) was used to purify DNA from loaded/non‐loaded bioengineered SkM when assessing the DNA methylation status of genes previously identified in Seaborne et al., (2018b, 2018a), including ODF2, UBR5, TRAF1, and GRIK2 (all sequencing information and raw data for these genes is presented in Supplementary File 3a,b). The AllPrep RNA/DNA Mini Kit was used to copurify RNA/DNA from muscle constructs when assessing DNA methylation of genes identified in Turner et al., (2019b), including MSN, WNT9a, GSK3β, TIMP3, and CTTN (all sequencing information and raw data for these genes is presented in Supplementary File 3c,d). After DNA extractions, concentrations and purities were quantified using a NanoDrop. Two‐hundred and fifty nanograms of extracted DNA/sample was bisulfite converted using the EZ‐96 DNA Methylation Kit (Zymo Research Corp.) as per the manufacturer's instructions. Bisulfite converted DNA was eluted in 46 µl of M‐elution buffer and amplified via multiplex PCR. Briefly, each 20 μl PCR reaction consisting of 0.5 U HotStarTaq Polymerase (Qiagen), 0.2 μM of forward and reverse primers for the gene of interest reported above and 2 μl of bisulfite‐treated DNA was amplified as follows: 95°C for 15 min (transcriptase inactivation and initial denaturation step) and then 45 × cycles of 95°C for 30 s (denaturation), 30 s annealing (the annealing temperature for each assay is described in Supplementary File 3), 72°C for 5 min (extension). After amplification, all PCR products were verified and quantified using the QIAxcel Advanced System (Qiagen). Before library preparation, PCR products from the same sample were pooled and purified using QIAquick PCR Purification Kit columns (Qiagen). Libraries were then prepared by EpigenDx (Hopkinton). Library molecules were purified using Agencourt AMPure XP beads (Beckman Coulter) and quantified using the Qiagen QIAxcel Advanced System. Template preparation and enrichment were performed using the Ion Chef system (Thermo Fisher Scientific) using Ion 520™ and Ion 530™. ExT Chef reagents (Thermo Fisher Scientific). Enriched template‐positive library molecules were sequenced using the Ion S5™ sequencer using an Ion 530™ sequencing chip (Thermo Fisher Scientific). FASTQ files from the Ion Torrent S5 server were aligned to the local reference database using the open‐source Bismark Bisulfite Read Mapper with the Bowtie2 alignment algorithm. Methylation levels were calculated in Bismark by dividing the number of methylated reads by the total number of reads (presented in Supplementary File 3b,d). If a data set displayed less than 30 reads, the results were considered unreliable and were therefore excluded from further analysis.

### 2.7 Acute resistance exercise in humans

To compare the transcriptional and epigenetic responses after loading in bioengineered SkM with those following RE in humans, data were compared to that already obtained in our group from eight healthy young (27.6 ± 2.4 years, 82.5 ± 6.0 kg, 178.1 ± 2.8 cm, means ± *SEM*) males who undertook a single bout of RE (Seaborne et al., 2018b, 2018a, 2019). Briefly, following a week of familiarization, untrained male participants performed an acute bout of RE consisting of several lower body exercises, including the back squat, leg press, leg extension, leg curl, Nordic curls, weighted lunges, and calf raises. Each exercise session consisted of 4 sets × 10 reps, ~90–120 s rest between sets and ~3 min rest after every 4 sets of 10 reps, totaling a regime of ~1 h. SkM biopsies were obtained at 0.5 h post exercise to determine changes in gene expression and DNA methylation. Ethical approval was granted by the NHS West Midlands Black Country, UK, Research Ethics Committee (NREC approval no. 16/WM/0103).

### 2.8 Programmed resistance training in rodent SkM

To compare the loading response in fibrin‐bioengineered muscle with the responses to resistance training (RT) in rodent SkM, adult (6 months) male Wistar rats were subject to programmed RT via intermittent high‐frequency (100 Hz) electrical stimulation as previously described (Schmoll et al., 2018; Seaborne et al., 2019). Briefly, under anesthesia with 2% inhaled isoflurane and buprenorphine I.M. injection at 0.1 mg/kg, rodents were implanted with a miniature stimulator sutured into the abdominal cavity with electrodes placed near the common peroneal and tibial nerves. The muscles were electrically stimulated to ensure that the dorsiflexors worked against the plantar‐flexors, and therefore the dorsiflexors were loaded. The stimulation consisted of an intermittent regime of high‐frequency (100 Hz) stimulation once a day for 4 weeks (5 sets × 10 reps, each repetition lasted 2 s with a 2 s rest between repetitions and 2.5 min rest between sets). This regime leads to a 14% and 19% increase in tibialis anterior (TA) muscle weight and fiber CSA, respectively (Schmoll et al., 2018; Seaborne et al., 2019). Following the cessation of electrical stimulation, RNA was isolated from the TA muscle of the stimulated and contralateral unstimulated (control) limbs (*n* = 5) using the TRIzol method as described above. Experimental procedures were conducted according to permissions within a project license granted under the British Home Office Animals (Scientific Procedures) Act 1986. Death was achieved by rising CO_2_ (100% CO_2_ at a flow rate of ~20% of chamber volume) followed by cervical dislocation.

### 2.9 Statistical analysis

A multivariate analysis of variance (MANOVA) was conducted using Minitab® software (Version 18) to determine main effects for multiple genes across conditions (loaded/non‐loaded) and time (0.5, 3 and 24 h) in bioengineered SkM. Unpaired *t* tests were conducted using GraphPad software (Prism, Version 7.0a) when assessing gene expression and DNA methylation between loaded and non‐loaded bioengineered muscle. A one‐way analysis of variance (ANOVA) with post hoc analysis (Tukey HSD) enabled detection of significant differences between loaded bioengineered SkM, human RE, and rodent RT for genes identified in Seaborne et al., (2018b, 2018a). A two‐way mixed ANOVA (2 × 3) was performed using Minitab® software (Version 18) to detect statistically significant interactions for condition (loaded/non‐loaded) and time (0.5, 3, and 24 h) when assessing genes that were significantly altered across the methylome and transcriptome after acute human RE in Turner et al., (2019b). Post hoc analysis (Tukey HSD) was carried out to confirm statistical significance between conditions (loaded/non‐loaded) and within time (0.5, 3, and 24 h) whenever significant interactions were observed. Pooled transcriptome analysis in Turner et al., (2019b) generated mean expression values across all pooled transcriptome studies in the literature when the first analysis was conducted (April 2019), rather than expression values for each individual study. Therefore, unpaired *t* tests were conducted following one‐way ANOVA analysis to determine where significant differences between models (loaded bioengineered muscle, human RE, and rodent RT) occurred when analyzing genes identified in Turner et al., (2019b). The alpha value of significance was set at *p* ≤ .05. All data are presented as the mean ± *SEM*.

## 3 RESULTS

### 3.1 Mechanical loading in bioengineered muscle elicits a mechano‐sensitive gene expression profile

**Figure 3:**
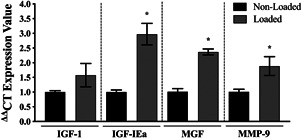
Gene expression of mechano‐sensitive genes after mechanical loading in mouse bioengineered SkM. Gene expression of IGF‐I, IGF‐IEa, MGF, and MMP‐9 at 3 h post‐loading in bioengineered SkM. *n* = 4 replicate cultures/constructs per condition (loaded/non‐loaded). *Represents the statistically significant increase in gene expression after mechanical loading (*p* ≤ .05). All data are presented as mean ± *SEM*. SkM, skeletal muscle

Previous work by our group first used the TC‐3 bioreactor system employed herein to assess gene expression of UBR5 after mechanical loading in bioengineered SkM (Seaborne et al., 2019). In the present study, to further characterize the bioreactor system and loading regime, mRNA expression of genes that are known to increase in bioengineered muscle after loading using already established/published bioreactor systems were investigated at 3 h post‐loading (Aguilar‐Agon et al., 2019; Cheema et al., 2005; Player et al., 2014). Mechanical loading of fibrin bioengineered SkM significantly increased mRNA expression of mechano‐sensitive genes IGF‐IEa (*p* = .01), MGF (*p* < .001), and MMP‐9 (*p* = .03; Figure 3). Thus, demonstrating that acute loading of fibrin bioengineered muscle using the TC‐3 bioreactor in the present study responded similarly to that previously observed using established bioreactor systems (Aguilar‐Agon et al., 2019; Cheema et al., 2005; Player et al., 2014).

### 3.2 Mechanical loading of bioengineered muscle recapitulates the transcriptional response observed at 0.5 h post‐acute RE in humans and programmed resistance training in rodents

**Figure 4:**
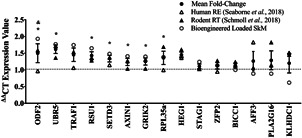
Gene expression following mechanical loading in bioengineered mouse SkM was compared with resistance exercise (RE) in human SkM and programmed resistance training (RT) in rodents. Genes in order of highest (ODF2) to lowest (KLHDC1) expression at 0.5 h post‐mechanical loading in bioengineered SKM alone. Clear circles represent gene expression at 0.5 h post‐loading in bioengineered SKM; clear triangles represent gene expression in human SKM at 0.5 h post‐acute RE in humans; bold triangles represent gene expression after programmed RT in rodents; bold circles with error bars represent mean ± *SEM* for the human, rodent, and bioengineered muscle. *Depicts significant increase in gene expression immediately post‐loading in bioengineered SKM compared with non‐loaded controls (*p* ≤ .05). ^&^Depicts significant difference in ODF2 gene expression between loaded bioengineered muscle and acute RE in humans (*p =* .004). All other genes demonstrated no significant differences between bioengineered mouse and human muscle. No significant differences were observed when comparing gene expression between bioengineered and rodent muscle after loading and RT, respectively. SkM, skeletal muscle

To investigate whether mechanical loading of bioengineered fibrin SkM recapitulates the transcriptional response to RE in vivo, we compared expression profiles of genes that were hypomethylated and upregulated after RE in humans (Seaborne et al., 2018a, 2018b) with gene expression 0.5 h after a single bout of intermittent mechanical loading in bioengineered muscle and programmed RT in rats (Schmoll et al., 2018; Seaborne et al., 2019). This included genes; UBR5, ODF2, RSU1, SETD3, GRIK2, RPL35a, AXIN1, TRAF1, STAG1, PLA2G16, KLHDC1, HEG1, AFF3, ZFP2, and BICC1. MANOVA analysis demonstrated there was a significant main effect for loading of bioengineered muscle for gene expression across all of these transcripts (*p* = .01), suggestive that the majority of genes were differentially expressed post‐loading in bioengineered muscle. Interestingly, the expression levels of these genes did not statistically differ between bioengineered and rodent muscle, evidenced by non‐significant differences observed between loading and RT in bioengineered and rodent SM, respectively (Figure 4). Moreover, 94% of genes (14/15 genes) demonstrated similar fold changes in gene expression in loaded bioengineered muscle and in human SkM. The remaining gene, ODF2, significantly increased in the mechanically loaded bioengineered muscle (*p* = .004; Figure 4) but did not significantly increase until after chronic RE in humans (Seaborne et al., 2018b, 2018a). Collectively, such findings suggest that the majority of genes were differentially expressed in a similar pattern for both loaded bioengineered muscle and after RE in humans. When assessing gene expression in bioengineered SKM alone, 47% of these genes significantly increased post‐loading compared with non‐loaded controls. This included genes; UBR5 (*p* = .01), ODF2 (*p* = .001), RSU1 (*p* = .01), SETD3 (*p* = .05), GRIK2 (*p* = .02), RPL35a (*p* = .05), and AXIN1 (*p* = .001; Figure 4).

### 3.3 Mechanical loading of bioengineered muscle only partially recapitulates the epigenetic response of human SKM after acute RE

**Figure 5:**
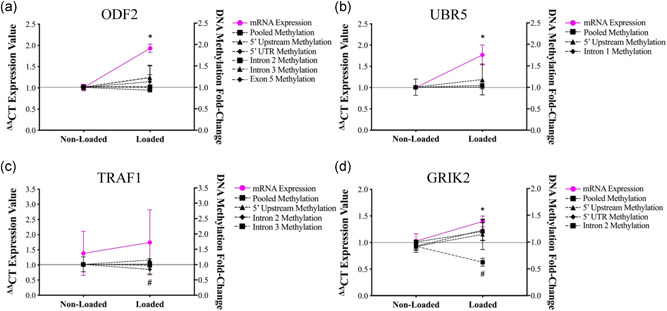
Gene expression and DNA methylation of genes identified to be upregulated and hypomethylated after RE in humans were assessed at 0.5 h post‐mechanical loading in bioengineered SKM. (a) ODF2 messenger RNA (mRNA) expression and DNA methylation was assessed at 0.5 h post‐loading (loaded) versus non‐loaded controls. *Depicts a significant increase in gene expression with no changes in DNA methylation (*p* ≤ .05). (b) Similarly, UBR5 mRNA expression also increased with no changes in gene expression. (c) TRAF1 gene expression did not significantly change. However, DNA methylation increased within the 5′ upstream region (^#^). (d) GRIK2 gene expression significantly increased (*), together with increased methylation within intron 2 (^#^). *n* = 4 replicate cultures/constructs per condition (loaded/non‐loaded). Data are presented as mean ± *SEM*. RE, resistance exercise; SkM, skeletal muscle

After assessing gene expression, we next analyzed DNA methylation of gene regulatory regions via targeted next‐generation bisulfite sequencing of the top 3 genes (ODF2, UBR5, TRAF1) that displayed the largest increases in gene expression post‐mechanical loading in the bioengineered muscle, which was also shown to have the corresponding hypomethylation after RE in humans (Seaborne et al., 2018a, 2018b). Despite the upregulation of gene expression observed after loading, DNA methylation of ODF2 and UBR5 did not significantly change (Figures 5a,b, respectively). In contrast to human muscle, TRAF1 was hypermethylated post‐acute loading, particularly in the 5’ upstream region (*p* = .02, Figure 5c). Finally, GRIK2 methylation was also assessed, given this gene significantly increased after loading in bioengineered SkM (Figure 4). In human muscle, GRIK2 methylation significantly reduced after a single bout of exercise, which was maintained approximately 22 weeks later throughout training, detraining, and retraining (Seaborne et al., 2018a, 2018b), suggestive of a potential epigenetic memory gene. In the present study, GRIK2 was also significantly hypomethylated, particularly in the intron 2 at 0.5 h post‐loading (*p* = .01; Figure 5d). Despite the reduction in GRIK2 methylation reported herein, DNA methylation profiles of the other genes analyzed did not closely mimic the response observed in human muscle.

### 3.4 Gene expression at 3 h post‐loading in bioengineered muscle recapitulates the transcriptional response to RE in human and rodent muscle

**Figure 6:**
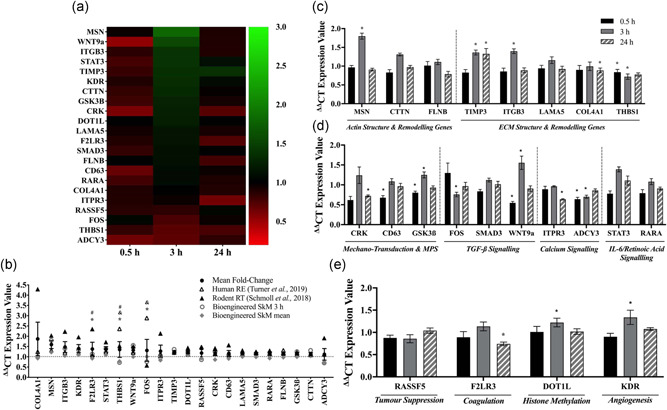
Gene expression of upregulated/hypomethylated identified in integrative methylome and transcriptome analysis (Turner et al., 2019b). (a) Heatmap representation of the temporal change in gene expression in bioengineered SkM at 0.5, 3, and 24 h post‐loading. Fold‐change was determined via relativizing gene expression in loaded to non‐loaded muscle for each separate timepoint (0.5, 3, or 24 h, as indicated on the *x*‐axis). The color intensity represents the level of fold‐change in gene expression (as indicated on the right *y*‐axis). Upregulated/hypomethylated genes are in order (from top to bottom) of the largest (MSN) to smallest (ADCY3) increase in gene expression at 3 h post‐loading. (b) Gene expression of upregulated/hypomethylated genes identified in Turner et al., (2019b) were compared between loaded bioengineered SkM at 3 h only (clear circles), when all timepoints (0.5, 3, and 24 h) were pooled (diamonds) and after acute RE in humans (clear triangles) and programmed RT in rodents (bold triangles). Bold circles with errors represent mean ± *SEM* when all models/timepoints of exercise/loading were pooled. *Indicates a significant difference between loaded bioengineered SKM at 3 h and human acute RE. ^&^Indicates significant difference between mean expression of pooled timepoints (0.5, 3, and 24 h) in loaded bioengineered SkM and human acute RE. ^#^Indicates significant difference between loaded bioengineered SKM and RT in rodents. Temporal gene expression profile (0.5, 3, and 24 h) in loaded versus non‐loaded bioengineered SkM alone for genes associated with (c) actin/ECM structure and remodeling (d) mechano‐transduction, muscle protein synthesis (MPS) and TGF‐β/calcium/IL‐6/retinoic acid signaling, and (e) tumor suppression, histone methylation, coagulation and angiogenesis identified in Turner et al., (2019b). *Depicts significant change in gene expression after loading relative to non‐loaded bioengineered muscle at the same timepoint. *n* = 5 replicate cultures/constructs per condition (loaded/non‐loaded) and timepoint (0.5, 3, and 24 h). All data is presented as mean ± *SEM*. IL, interleukin; RE, resistance exercise; RT, resistance training; SkM, skeletal muscle; TGF, tumor growth factor

In an attempt to identify whether mechanical loading of bioengineered muscle mimicked the temporal gene expression response in human SkM over a longer time‐course post‐acute RE, we next analyzed mRNA expression of genes that were significantly upregulated across the majority of published transcriptome data sets (total 110 biopsies) that included biopsies obtained immediately and up to 24 h post‐acute RE in human SKM (Turner et al., 2019b) that were also significantly hypomethylated post‐acute exercise in Seaborne et al., (2018b, 2018a). Gene expression of the same genes was therefore analyzed at 0.5, 3, and 24 h post‐acute mechanical loading in bioengineered mouse SkM. Upregulated/hypomethylated genes from the pooled transcriptome analysis in Turner et al., (2019b) included 22 genes associated with ECM/actin structure and remodeling (MSN, CTTN, FLNB, TIMP3, ITGB3, LAMA5, COL4A1, THBS1), mechano‐transduction (CRK, CD63), protein synthesis (GSK3β) and TGF‐β (FOS, SMAD3, WNT9A), calcium (ITPR, ADCY3), IL‐6 (STAT3), retinoic acid (RARA) signaling, coagulation (F2LR3), angiogenesis (KDR), histone methylation (DOT1L), and tumor suppression (RASSF5). Heatmap analysis of gene expression over the time‐course post‐loading in bioengineered muscle suggested that gene expression was predominantly differentially regulated at 3 h (Figure 6a). Plotting the mean expression of the 22 upregulated/hypomethylated genes over 24 h in Turner et al., (2019b) against the 3 h and mean expression values (0.5, 3, and 24 h pooled) in loaded bioengineered SkM demonstrated that 82% (18 out of 22) of these genes did not statistically differ between loaded bioengineered and human muscle (Figure 6b). These 18 genes included MSN, CTTN, FLNB, TIMP3, ITGB3, LAMA5, COL4A1, CRK, CD63, GSK3β, SMAD3, WNT9A, ITPR, STAT3, RARA, F2LR3, KDR, and DOT1L. Only four genes were statistically dissimilar between loaded bioengineered mouse muscle and human muscle after acute RE (RASSF5 *p* = .04, THBS1 *p* < .001, ADCY3 *p =* .03, FOS *p* = .001). Moreover, a comparison of gene expression signatures between loaded bioengineered and rodent muscle tissue revealed that 91% (20 out of 22) of these genes were similar, with the remaining 2 genes displaying significantly higher expression in rodent muscle tissue (F2LR3 *p* = .02, THBS1 *p* = .003). When comparing gene expression between loaded and non‐loaded bioengineered muscle alone, 45% of the genes that were upregulated across the transcriptome in Turner et al., (2019b) also increased at 3 h post‐loading. This included six genes (MSN, TIMP3, ITGB3, GSK3β, WNT9a, KDR) that were significantly upregulated and an additional four genes (STAT3, DOT1L, CRK, CTTN) that showed a modest non‐significant increase. A finding that suggested loading in bioengineered muscle was able to elicit similar expression patterns of genes identified across the majority of published transcriptome datasets post‐acute RE in humans (Turner et al., 2019b), at least at 3 h. It is worth noting that three (THBS1, FOS, ADCY3) of the upregulated genes in Turner et al., (2019b) showed a reduction at 3 h, with the remaining genes demonstrating no significant changes. Interestingly, significant increases at 3 h post‐loading were observed in the actin structure and remodeling gene MSN (*p* < .001; Figure 6c). Out of the genes related to ECM structure and remodeling, TIMP3 and ITGB3 also significantly increased at 3 h (*p* = .02 and *p* = .01, respectively; Figure 6c). In mechano‐transduction/MPS, TGF‐β signaling and angiogenesis associated genes; GSK3β, WNT9a (Figure 6d), and KDR (Figure 6e) expression was also greater after loading in bioengineered muscle at 3 h (*p* = .01, *p* = .001, *p* = .03, respectively). While there were significantly increased gene expression at 3 h post‐loading for these genes, no genes were upregulated at 0.5 h post‐loading, albeit five genes (THBS1, CD63, GSK3β, WNT9a, ADCY3) showed an acute reduction in gene expression (Figure 6c–e). Finally, one gene (TIMP3) remained elevated at 24 h post‐loading whereas four genes (COL4A1, CRK, ITPR3 F2LR3) showed a reduction in gene expression (Figure 6c–e), suggestive that mechanical loading in the bioengineered muscle did not lead to increases in overall gene expression at earlier (0.5 h) and later (24 h) timepoints. It is also worth noting that analysis of human muscle involved pooling transcriptomic datasets across 110 biopsies (37 pre/57 post after outlier removal) over multiple studies (Turner et al., 2019b), and therefore encompassed all timepoints from immediately post and up to 24 h post‐acute RE. Where, gene expression is typically greatest at 3–8 h post exercise (Barrès et al., 2012; Chen et al., 2002; Drummond et al., 2008; Knuiman et al., 2018; Kuang et al., 2020) and generally returns to basal levels within 24 h (Egan & Zierath, 2012; Liu et al., 2010; Yang et al., 2005). Therefore, the majority of transcriptional alterations detected at 3 h in the bioengineered muscle may also be exemplifying this temporal activation in gene expression post‐loading.

### 3.5 Epigenetic changes in bioengineered SKM post‐loading at 3 h do not mimic the changes observed in humans after acute RE

**Figure 7:**
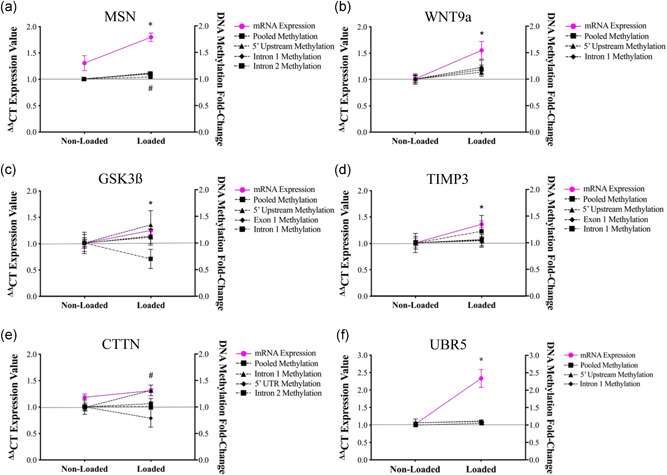
DNA methylation of genes that were upregulated and hypomethylated after acute RE in Turner et al., (2019b) were assessed at 3 h post‐loading in bioengineered SKM. (a) MSN. *Depicts significant increase in gene expression and ^#^intron 1 and pooled methylation at 3 h post‐loading. *Depicts significant increase in gene expression for (b) WNT9a, (c) GSK3β, and (d) TIMP3. (e) CTTN. ^#^Depicts a significant increase in intron 1 methylation. (f) UBR5. *Depicts significant increase in gene expression at 3 h post‐loading. *n* = 5 replicate cultures/constructs per condition (loaded/non‐loaded). Data is presented as mean ± *SEM*. RE, resistance exercise; SkM, skeletal muscle

Given that the transcriptional responses for genes identified in Turner et al., (2019b) were comparable between acute RE in human muscle and mechanical loading in bioengineered SKM at 3 h, we next sought to investigate the DNA methylation response of several of these genes at 3 h post‐loading. This would identify whether mechanical loading also mediates corresponding DNA methylation changes of the same gene that were reported in human muscle post‐acute RE. Specifically, DNA methylation analysis was conducted in gene regulatory regions for the most upregulated genes (MSN and WNT9a) at 3 h post‐loading. TIMP3 and GSK3β DNA methylation was also analyzed as these genes also significantly increased at 3 h post‐loading and are known important regulators of ECM remodeling and muscle protein synthesis, respectively. Finally, DNA methylation of CTTN was also assessed, despite demonstrating a modest non‐significant increase post‐loading. This was due to its known importance for actin cytoskeleton remodeling and, together with FLNB, was one of the only genes that was significantly upregulated and hypomethylated after both acute and chronic RE in human muscle (Turner et al., 2019b). We report no significant change in MSN intron 2 (*p* = .09) or 5’ upstream (*p* = .06) region‐specific DNA methylation at 3 h post‐loading (Figure 7a). DNA methylation of MSN actually increased, particularly in the intron 1 region (*p* < .001) and when intron 1/2 and 5’ upstream regions were pooled (*p* < .001; Figure 7a). For genes, WNT9a, GSK3β, and TIMP3, there was no significant change in DNA methylation at 3 h post‐loading for all regions assessed (Figure 7b–d). In contrast to human muscle post‐RE in vivo, CTTN methylation significantly increased, particularly in intron 1 (*p* = .01; Figure 7e). As these genes all demonstrated reduced hypomethylated signatures after acute RE in humans, data reported herein demonstrate only a minor congruence with the changes observed after acute RE in humans (Turner et al., 2019b). Finally, given UBR5 hypomethylation and increased gene expression has been extensively characterized under various anabolic conditions across species (Hughes et al., 2020; Seaborne et al., 2018b, 2018a, 2019) together with demonstrating the largest increases in gene expression compared with any other gene analyzed in the present study, we analyzed its DNA methylation at both 0.5 h (described above) and 3 h post‐loading. Despite robust increases in gene expression at both 0.5 h (1.77‐fold) and 3 h (2.34‐fold) postloading, there was no change in DNA methylation observed at 0.5 h (described above in Figure 5b) and 3 h post‐loading in the bioengineered muscle (Figure 7f). Collectively, data presented herein suggest that mechanical loading in the bioengineered muscle was unable to sufficiently recapitulate the DNA methylation responses observed for genes known to be upregulated/hypomethylated after acute RE in humans (Turner et al., 2019b).

## 4 DISCUSSION

The present study aimed to determine whether mechanical loading of C_2_C_12_ fibrin bioengineered SkM in vitro recapitulates the transcriptional and DNA methylation signature of human and rodent SkM after RE in vivo. Data presented herein suggests that mechanical loading of mouse bioengineered SkM in vitro induced comparable transcriptional responses to human and rodent SKM after RE in vivo. However, while some DNA methylation modifications were detected following loading, such changes did not closely mimic the DNA methylation response to acute RE in humans.

The present study first sought to validate whether mechanical loading of mouse bioengineered SkM using the bioreactor employed herein was able to evoke a mechano‐transcriptional response, similar to that previously observed using well‐established bioreactor systems. Indeed, increases in mechano‐sensitive genes, IGF‐IEa, MGF, and MMP‐9, together with a modest increase in IGF‐I, were observed as previously reported following loading in the collagen‐matrix bioengineered muscle (Aguilar‐Agon et al., 2019; Cheema et al., 2005; Player et al., 2014). To directly compare the transcriptional and DNA methylation response to mechanical loading in mouse bioengineered muscle with that observed after acute RE in human and rodent muscle, mRNA expression of genes that have been recently identified to demonstrate differential methylation (following unbiased analysis of genome‐wide DNA methylation) and corresponding alterations in gene expression post RE in humans (Seaborne et al., 2018a, 2018b) were analyzed after loading. Interestingly, 93% (14 out of 15 genes, including UBR5, ODF2, RSU1, SETD3, GRIK2, RPL35a, AXIN1, TRAF1, STAG1, PLA2G16, KLHDC1, HEG1, AFF3, ZFP2, and BICC1) of these genes demonstrated no significant differences in gene expression between loaded mouse bioengineered muscle and human muscle at 0.5 h post‐loading and RE, respectively. Moreover, no differences were observed between loaded bioengineered muscle and programmed RT in rodents for any of these genes. Overall, suggesting similar gene expression changes in mechanically loaded bioengineered muscle to that observed in human and rat muscle after RE in vivo. In an attempt to identify whether mechanical loading of bioengineered muscle mimicked the temporal gene expression profile after acute RE in humans over a longer timecourse, mRNA expression of genes that were upregulated across published transcriptome data sets post‐acute RE in humans (Turner et al., 2019b) with the corresponding hypomethylation of the same genes (Seaborne et al., 2018b, 2018a) were analyzed in loaded bioengineered SkM. These genes were therefore analyzed at 0.5, 3, and 24 h post‐loading in bioengineered mouse muscle to enable a direct comparison with humans after acute RE. First, the majority of these genes predominantly increased at 3 h post‐loading and returned to basal levels at 24 h. This temporal gene regulatory profile has been observed several times in response to exercise in vivo in which gene expression tends to peak at 3–8 h post‐exercise (Barrès et al., 2012; Chen et al., 2002; Drummond et al., 2008; Knuiman et al., 2018; Kuang et al., 2020) and generally returns to basal levels within 24 h (Egan & Zierath, 2012; Liu et al., 2010; Yang et al., 2005). When comparing the changes in gene expression at 3 h with the human transcriptome data, 83% of these genes (MSN, CTTN, FLNB, TIMP3, ITGB3, LAMA5, COL4A1, CRK, CD63, GSK3β, SMAD3, WNT9A, ITPR, STAT3, RARA, F2LR3, KDR, DOT1L) showed no significant difference in expression between loaded mouse bioengineered muscle and human muscle after acute RE. Gene expression for these genes was also compared between loaded bioengineered muscle and RT in rodents in vivo. Interestingly, only 2 out of 22 genes were statistically different, suggestive that 91% responded similarly between bioengineered and rodent muscle tissue. Overall, together with the aforementioned data above, these results suggest that mechanical loading in mouse bioengineered muscle sufficiently recapitulates the transcriptional response of SkM following RE in vivo.

It is also worth highlighting that the E3 ubiquitin ligase, UBR5, was the most upregulated gene at 3 h post‐loading in bioengineered muscle alone compared with any gene analyzed in the present manuscript, significantly increased by 1.77‐fold at 0.5 h and 2.34‐fold at 3 h. Interestingly, this HECT domain E3 ubiquitin ligase was hypomethylated and upregulated after acute and chronic RE in untrained human participants, with enhanced hypomethylation and gene expression after later retraining (Seaborne et al., 2018a, 2018b, 2019). Such alterations were positively correlated with changes in lean mass (Seaborne et al., 2018a, 2018b, 2019). In rodent muscle, recent work has also confirmed that UBR5 gene expression and protein levels increase in response to hypertrophy in vivo, with no changes observed in the well‐characterized atrogene E3 ligases, MuRF1 and MAFbx (Seaborne et al., 2019). Moreover, its role in muscle mass regulation has been recently determined whereby RNAi induced silencing in *Drosophila* results in smaller sized larvae (Hunt et al., 2019) and RNAi electroporated into mouse TA muscle in vivo leads to atrophy via reduced protein synthesis and dysfunctional ERK/Akt signaling (Hughes et al., 2020). Collectively, such data support the notion that UBR5 is important for load‐induced anabolism and hypertrophy. Interestingly, UBR5 gene expression increased to a similar extent at 0.5 h post‐loading in C_2_C_12_ bioengineered SkM (1.77‐fold) with that observed post‐acute RE in human SkM tissue (~1.7‐fold; Seaborne et al., 2018a, 2019), following programmed RT in rats (1.5‐fold; Seaborne et al., 2019) and at 3 h post mechanical loading in human myotubes in monolayer (1.6‐fold, unpublished data by our group). However, no corresponding hypomethylation was observed after loading in bioengineered muscle in the present study. Indeed, GRIK2 was the only gene that was significantly hypomethylated after loading. Such findings are interesting given GRIK2 was significantly hypomethylated after a single bout of exercise in vivo, which was maintained ~22 weeks later throughout training, detraining, and retraining (Seaborne et al., 2018b), suggestive of a epigenetic memory signature. Furthermore, recent methylome analysis of acute overload in mouse plantaris muscle revealed intron region‐specific hypomethylation of GRIK3 and GRIK4 genes in myonuclei and interstitial cells, respectively (Von Walden et al., 2020), suggestive of a cell‐specific role of GRIK family genes in response to exercise/loading. Finally, TRAF1, MSN, and CTTN were significantly hypermethylated, which was in contrast to the hypomethylation observed in human muscle. The lack of DNA methylation changes, however, is interesting given that the transcriptional program was still similar. One explanation may involve the requirement for concentric contractions as the model employed herein resembles eccentric lengthening of the bioengineered muscle only. Therefore, these data may suggest that neural input induced by concentric contraction may be a more potent driver of DNA methylation perturbations in response to exercise in vivo. Indeed, active concentric contractions require substantial cycling of cytosolic calcium concentrations. This calcium signal could drive the phosphorylation of methyl CpG‐binding protein 2 (MeCP2) associated with the induction of alterations in DNA methylation (reviewed in Seaborne & Sharples, 2020). The absence of secretory products from other cell types within the C_2_C_12_ bioengineered muscle constructs may also partially explain the differential epigenetic response to RE in vivo as recent work reports that communication between extracellular vesicles and the myofiber influences the response to loading in mouse muscle (Murach et al., 2020). To challenge this hypothesis, future studies, perhaps with the inclusion of electrical stimulation to drive concentric contraction and use of rodent (Khodabukus & Baar, 2015c) or human primary muscle‐derived cells (Martin et al., 2013) would be an important avenue of research (reviewed in Kasper et al., 2018).

## 5 CONCLUSION

Mechanical loading of mouse bioengineered SKM in vitro recapitulates the gene expression profiles of SkM after REg in vivo. However, while some DNA methylation changes were detected following mechanical loading in bioengineered muscle, this did not as closely mimic the DNA methylation response to acute RE in vivo.

## CONFLICT OF INTERESTS

The authors declare that there are no conflict of interests.

## AUTHOR CONTRIBUTIONS

Daniel C. Turner and Adam P. Sharples conceived and designed the research. All authors were involved in the acquisition or analysis or interpretation of data for the work. Daniel C. Turner and Adam P. Sharples drafted the work. All authors were involved in revising the work critically for important intellectual content. All authors approved the final version of the manuscript.

## Supporting information

Supporting information.

Supporting information.

Supporting information.

## Data Availability

The authors declare that the data supporting the findings of the present study are available within the manuscript or from the corresponding authors upon reasonable request.
