# Supplementary figures and images for: Mechanical loading of bioengineered skeletal muscle in vitro recapitulates gene expression signatures of resistance exercise in vivo
Source: J Cell Physiol. 2021 Feb 15;236(9):6534–47. doi: 10.1002/jcp.30328 (PMC8653897; doi:10.1002/jcp.30328)

**Supplementary File 1. Video of C2C12 bioengineered SkM spontaneously twitching at day 14**
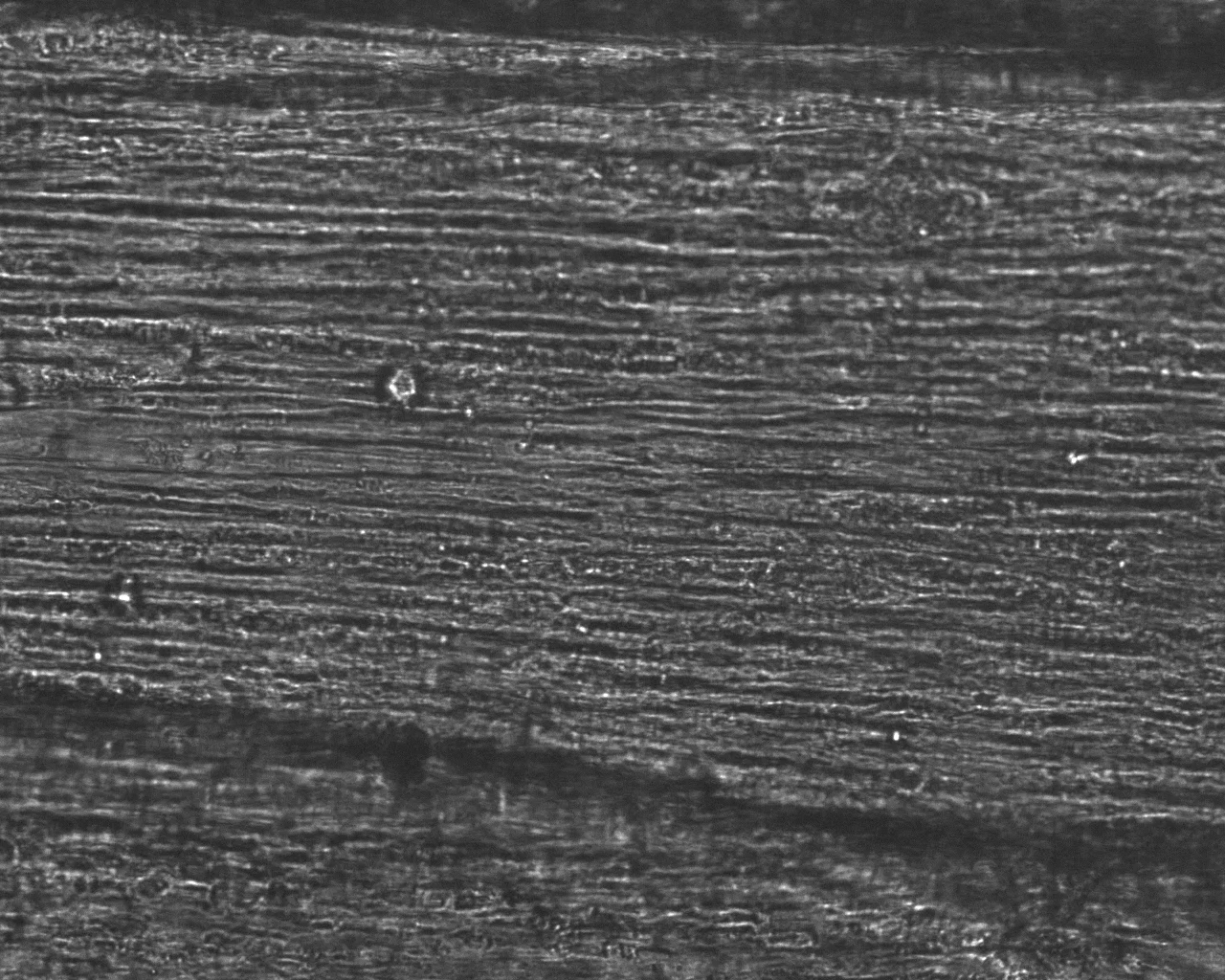

Supplement: Supplementary file 1 — Supporting information. [file JCP-236-6534-s002.docx]
