# Supplementary material for: Mechanical loading of bioengineered skeletal muscle in vitro recapitulates gene expression signatures of resistance exercise in vivo
Source: J Cell Physiol. 2021 Feb 15;236(9):6534–47. doi: 10.1002/jcp.30328 (PMC8653897; doi:10.1002/jcp.30328)
Supplement: Supplementary file 2 — Supporting information. [file JCP-236-6534-s001.pdf]

Supplementary File 2A.
Mouse primers for assessing mechano-responsive genes after loading in bioengineered SkM

Target Gene	Primer Sequence (5'-3' end)	Product Length (bp)	Reference Sequence Number
IGF-1	F: CACACCTCTTCTACCTGGCG	189	NM_001314010.1
	R: CCACAATGCCTGTCTGAGGT		
IGF-1Ea	F: GCTTGCTCACCTTTACCAGC	300	NM_010512
	R: AATGTACTTCCTTCTGGGTCT		
MGF	F: GCTTGCTCACCTTTACCAGC	353	NM_010512.5
	R: AAATGTACTTCCTTTCCTTCTC		
MMP-9	F: CTGGCAGAGGCATACTTG	76	NM_013599.2
	R: GCCGTAGAGACTGCTTCT		
RP-IIß	F: GGTCAGAAGGGAACTTGTGGTAT	197	NM_153798.2
	R: GCATCATTAAATGGAGTAGCGTC		

Supplementary File 2B.

Mouse primer sequences for assessing genes that significantly changed across the human methylome after RE in humans (Seaborne *et al.*, 2018*b*, 2018*a*)

Target Gene	Primer Sequence (5'-3')	Product Length (bp)	Reference Seq. Number
UBR5	F: GTCTGCTGGAGCTCGTGATT	106	XM_006520182.3
	R: TGCTGGAATAACTGGCTGGG		XM_006520181.3
			XM_006520180.3
			XM_006520179.3
			NM_001081359.3
			NM_001112721.2
AFF3	F: TCGCCGCCTCCACTAATAAC	196	XM_011238447.2
	R: GAAGTCAACAACCCGTTGCC		XM_006495737.3
			XM_011238446.2
			XM_011238444.2
			XM_011238443.2
			NM_001290814.1
ODF2	F: TTGCACCGACATCAACACCT	114	NM_001355137.1
	R: TTGCAGTGCTGTTCCCTCAA		NM_001355136.1
			NM_001355138.1
			XM_017316304.1
			XM_017316300.1
			XM_017316299.1
			XM_017316295.1
			XM_017316294.1
			XM_017316293.1
			XM_017316292.1

			XM_017316291.1
			XM_017316290.1
			XM_017316288.1
			XM_017316286.1
			XM_017316285.1
			XM_017316284.1
			XM_017316283.1
			XM_017316281.1
			XM_017316280.1
			XM_017316279.1
			XM_017316276.1
			XM_017316275.1
			XM_017316273.1
			XM_017316272.1
			NM_001177661.1
			NM_001177659.1
			NM_001113214.1
			NM_001113213.1
			NM_013615.3
AXIN1	F: ACAGGATCCGTAAGCAGCAC	111	XM_006523516.2
	R: CCCGGATCTCCTTTGGCATT		XM_006523515.3
			NM_001159598.1
			NM_009733.2
RSU1	F: AACCCCTAGCAGCCAAGAAC	118	XM_006497405.3
	R: GAAAAGTAGGCACCAGCACG		NM_009105.4

HEG1	F: GAACGTAGAACGGGATGCCT	189	XM_017317159.1
	R: GCTTGGAAATGAGCACGGAC		XM_006522718.2
			XM_006522715.3
			XM_006522716.1
			NM_175256.5
TRAF1	F: AGCATGCTGGTTATGGCTGA	193	XM_011239054.2
	R: GGCTCAATGTCCAAGCCTCA		XM_011239053.2
			XM_011239052.2
			XM_017317129.1
			XM_011239051.2
			XM_011239050.2
			NM_009421.4
			NM_001326601.1
SETD3	F: GGGTGACCTTGGCTCTGATT	91	NM_001364267.1
	R: CAGCGGTCATCTTCCAGGTT		NM_001364269.1
			NM_001364266.1
			XM_006516078.3
			NM_028262.3
			XM_011245945.1
			NM_001081217.1
GRIK2	F: CAGCACTGGTCTCATTCGCT	84	NM_001358866.1
	R: GCTGGCAGCTGACGAATTTT		NM_010349.3
			NM_001111268.2
			XM_011243127.2
			XM_006512545.3

			XM_011243125.2
			XM_011243124.2
			XM_011243123.2
			XM_011243122.2
			XM_011243121.2
			XM_011243120.2
			NM_001093749.3
PLA2G16	F: GAATGACTGCCCAGTTTTTGGG	196	NM_001362425.1
	R: TGTTATCCAGAACAGAGCCCC		NM_139269.2
RPL35a	F: TATGCCCGAGATGAAACGGAG	171	NM_001130485.1
	R: GCTTCGGAATTTGGCACGAA		NM_001130484.1
			NM_021338.3
ZFP2	F: TGTGGGAAAACCTTCAGGCA	115	XM_017314508.1
	R: GGGAGGAGCGTTCGATGAAA		XM_017314507.1
			XM_017314506.1
			NM_178447.3
			NM_001044700.2
			NM_001044698.2
			NM_001044697.2
			NM_001294323.1
BICC1	F: TGGTAGCGGTACCTTCTGGA	75	NM_001347189.1
	R: TGCAGTGAAGACTGTCCACG		NM_031397.3
STAG1	F: GGGGTACTGGCAAGAGAGTG	198	NM_001357265.1
	R: TTTCCATCCGACCTGTGCTG		NM_001357264.1
			NM_009282.4

			XM_011242692.2
			XM_006510925.2
			XM_006510924.2
			XM_006510923.2
			NM_008636.4
KLHDC1	F: CACACAGAGACGGGTTTGGA	173	XM_011244121.2
	R: TGGCTTCTGATCAACGGAGG		XM_011244119.2
RP-IIß	F: GGTCAGAAGGGAACTTGTGGTAT	197	NM_153798.2
	R: GCATCATTAAATGGAGTAGCGTC		

Supplementary File 2C.

Mouse primer sequences for assessing genes identified in Turner *et al.*, (2019*b*) that were upregulated/hypomethylated after acute RE in humans

Target Gene	Primer Sequence (5'-3')	Product Length (bp)	Reference Seq. Number
MSN	F: GGATGCCTTGGGTCTCAACA	75	NM_010833.2
	R: ATTTCACTCCACGGGAAGCC		
TIMP3	F: CAAGGGCCTCAATTACCGCT	107	NM_011595.2
	R: TGTCGGTCCAGAGACACTCA		
WNT9a	F: CTGGCCTCCTCAACCCTTTT	75	NM_139298.2
	R: GCATTACTGCAACGCTCTCG		XM_006532855.2
CTTN	F: AGCATGCCTCCCAGAAAGAC	70	NM_001357116.1
	R: TCTACACGGTCAGCTTGCAC		XM_006508475.2
			XM_006508474.2
			NM_007803.5
			NM_001252572.1
GSK3ß	F: GAAGACTTGCCTTTGGCGTG	100	NM_019827.7
	R: TAGTGACCTCCCTGGGCTAC		NM_001347232.1
			XM_006522426.3
			NM_177322.3
FOS	F: TACTACCATTCCCCAGCCGA	113	NM_010234.3
	R: GCTGTCACCGTGGGGATAAA		
THBS1	F: TGTAAAGCCTGAGACCTGCC	71	NM_001313914.1
	R: TTCGTTAAAGGCCGAGTGCT		NM_011580.4
ITPR3	F: TGTCTGACCAGAAGAACGCC	85	XM_006523712.3
	R: TTGCGGTAGTCCTCCTGAGA		NM_080553.3
RARA	F: ATCTGTGGAGACCGACAGGA	96	NM_001361954.1

	R: CCGTTTCCGGACGTAGACTT		XM_006532592.3
			XM_006532597.2
			XM_006532593.2
			XM_006532593.2
			NM_001177302.1
			NM_001176528.1
			NM_009024.2
FLNB	F: AGTGCGATGCCCGAGTTTTA	104	NM_001081427.1
	R: AGGGGCAGGAGGTACGTATT		XM_006518050.1
			NM_134080.1
LAMA5	F: CGGGTATCAACTGTGAGCGT	71	XM_017315832.1
	R: ATGAGGTGAGTCGAGAGGCT		XM_017315831.1
			XM_006500575.2
			NM_001081171.2
RASSF5	F: TCCATACCCTTTCCTCGGGT	73	NM_001311094.2
	R: CTGGGCTGGTAGGGAACTTT		NM_018750.4
			NM_001313731.1
CRK	F: GCGTCTCCCACTACATCATCA	106	XM_006532125.2
	R: TCTCCTATTCGGAGCCTGGA		XM_006532124.2
			NM_133656.5
			NM_001277219.1
SMAD3	F: CGTGGAGTATGTGTCCTGGG	80	XM_006510821.3
	R: TACATCAGGGTTGTGGTGCC		XM_006510819.2
			NM_016769.4
STAT3	F: CACATGCCACGTTGGTGTTT	73	XM_017314401.1

	R: GACTCTTGCAGGAATCGGCT		XM_011248846.2
			NM_011486.5
			NM_213660.3
			NM_213659.3
COL4A1	F: GGCTCTCCGGGTTCAATAGG	84	XM_017312555.1
	R: GCCGATGTCTCCACGACTAC		NM_009931.2
ITGB3	F: CTGCCGGAAGAACTGTCACT	93	XM_006532312.2
	R: TCCAATCTTGAGGCCCACAC		NM_016780.2
KDR	F: TTTCACCTGGCACTCTCCAC	93	NM_001363216.1
	R: AACATCTTCGCCACAGTCCC		NM_010612.3
			XM_011240817.2
ADCY3	F: TTTATGCGGCTGACCTTCGT	70	XM_006514935.3
	R: GGCGCTGCCTTTTGAAGTAG		XM_006514934.3
			XM_006514933.2
			XM_006514932.2
			NM_001159537.1
			NM_001159536.1
			NM_138305.3
CD63	F: TTGAAGCAGGCCATTACCCA	71	NM_001282966.1
	R: GCACCCACTGCAATGATGAC		NM_001042580.1
			NM_007653.3
DOT1L	F: AAGGGAGAAAGATGGCTGGC	76	NM_199322.2
	R: TTAGACTTGCGTTCGGCACT		XM_006513432.3
			XM_011243391.2
			XM_006513429.3

			XM_011243390.2
			XM_006513431.1
			XM_006513430.1
			XM_006504845.2
			NM_001291248.1
			NM_001291249.1
			NM_178576.3
F2RL3	F: ACGCCTCACTACTGGACTCT	100	NM_007975.4
	R: GGAGCCAGCTAATCGGAAGG		XM_006530660.3
RP-IIß	F: GGTCAGAAGGGAACTTGTGGTAT	197	NM_153798.2
	R: GCATCATTAAATGGAGTAGCGTC		
